# Layered Location-Based Security Mechanism for Mobile Sensor Networks: Moving Security Areas

**DOI:** 10.3390/s151024886

**Published:** 2015-09-25

**Authors:** Ze Wang, Haijuan Zhang, Luqiang Wu, Chang Zhou

**Affiliations:** School of Computer Science and Software Engineering, Tianjin Polytechnic University, Tianjin 300387, China; E-Mails: zhanghaijuan113@outlook.com (H.Z.); luqiangwuwind@gmail.com (L.W.); zhouc1993@sina.com (C.Z.)

**Keywords:** mobile sensor networks, malicious attacks, location-based key, replication attack

## Abstract

Network security is one of the most important issues in mobile sensor networks (MSNs). Networks are particularly vulnerable in hostile environments because of many factors, such as uncertain mobility, limitations on computation, and the need for storage in mobile nodes. Though some location-based security mechanisms can resist some malicious attacks, they are only suitable for static networks and may sometimes require large amounts of storage. To solve these problems, using location information, which is one of the most important properties in outdoor wireless networks, a security mechanism called a moving security area (MSA) is proposed to resist malicious attacks by using mobile nodes’ dynamic location-based keys. The security mechanism is layered by performing different detection schemes inside or outside the MSA. The location-based private keys will be updated only at the appropriate moments, considering the balance of cost and security performance. By transferring parts of the detection tasks from ordinary nodes to the sink node, the memory requirements are distributed to different entities to save limited energy.

## 1. Introduction

Sensor networks, which usually comprise plenty of tiny sensor nodes with limited resources, have been demonstrated to be useful in most applications, such as battlefield communication and emergency search-and-rescue scenarios. Due to the advancement of robotics and microchip technology, a sensor node may also possess moving capability. Mobile sensor networks (MSNs) are composed of sensor nodes with moving capability. The moving characteristic of a mobile sensor node, *i.e.*, the mobility model, is usually described as change in position with an upper speed limit and moving trajectory. Taking into consideration nodes’ mobility, it is desirable, but more challenging, to design efficient and effective schemes against attacks in mobile sensor networks.

One of the most classic malicious attacks is a replication attack. Under this kind of attack, one node could be captured and many replicas of the compromised node could be made by using the same ID. These malicious nodes could get some information by accessing the network. Most communication protocols rely heavily on message forwarding in MSNs. In a wormhole attack, these replicas of the compromised node may collude with each other to destroy the communication protocols. This makes it difficult to ensure network security.

In most realistic scenarios, nodes constantly keep moving in the network, and some significant areas might require higher security levels than other areas. The proposed mechanism in this paper satisfies the above two requirements at the same time. Detailed illustrations will be provided in Section 4.

The concept of a location-based key (LBK) [1] was first put forward in 2006. A LBK is used in static wireless networks. In this situation, each node has a private key which is bound to its ID and geographic location to detect attacks. Since then, the location information of nodes has gradually become a crucial factor for MSNs. Some schemes have exploited the parameter related to the location information [2,3,4,5,6] to resist replication attacks.

Though the mechanisms based on LBK could resist some kinds of attacks, they are not suitable for dynamic networks. The positions of nodes are changing all the time in dynamic networks, so a LBK might be illegitimate at some point.

Xing K. [3] proposed a mechanism for detecting a replication attack by using the location information of nodes. Nodes exchange their neighbor lists to find out contradictions. Every node plays two roles: Ordinary node and witness node. This mechanism is successfully used in TDD and SDD to resist replication attacks. Much later, a localized mechanism [6] was proposed to resist replication attacks, but it is only used in dynamic networks.

The previous studies may only be suitable for a single network environment and often require large amounts of storage. To address many of these problems, this paper presents a layered location-based security mechanism for MSNs.

This paper aims to develop a security mechanism for MSNs. Nodes will not update their location-based private keys until moving out of the special areas, which are called moving security areas (MSA). What is more, the parameter Δ, which is the radius of the circular area MSA, can be regulated to satisfy different security needs. The main contributions from our work are as follows.

Firstly, we design a scheme in which the locations of nodes are embedded into their location-based public keys, so that the locations can be authenticated to resist location-based malicious attacks.

Secondly, we analyze the effective range of the location-based keys of mobile nodes, design the update scheme of the location-based keys, and resolve the problem that location-based keys are difficult to apply in mobile sensor networks.

Thirdly, a layered detection framework is proposed in our scheme. Inside the effective moving security areas of the nodes’ location-based keys, the detections could be executed by exchanging the nodes’ local information. Outside the moving security areas, the consistency of the mobile nodes’ locations could be detected by a sink node. On one hand, the heavy overheads on the sink node to detect attacks could be reduced, and on the other hand the detection effect of the local method could be improved.

Finally, we build a mathematical model to analyze the detection rate of the local detection scheme and prove the validity of the scheme. Moreover, we also verified the performance of the scheme through simulation experiments.

The remaining part of this paper is organized as follows. Section 2 illustrates the network model and security model. Section 3 details the proposed MSA mechanism. Section 4 analyzes the security and the cost of the proposed mechanism and then the conclusion is given in Section 5.

## 2. Models and Assumptions

### 2.1. Network Model

We assume the sensor network is composed of *N* sensor nodes, randomly deployed in a square area. The communication range of each node is denoted by *R*. There is a sink node which acts as the base station to collect data from the network. We assume that the minimum and the maximum moving speed are ${\text{V}}_{min}$ and ${\text{V}}_{max}$, respectively. The clocks of all nodes are assumed to be loosely synchronized. Assume that GPS systems or localization schemes proposed in [7,8,9] have been applied to the network such that all nodes know their own positions. Also, only some beacon nodes are equipped with GPS systems or other location support systems to be aware of their own locations. A distributed localization algorithm is executed in other common sensor nodes by using the location information of beacons to make them aware of their own location information.

In similar literature focusing on defeating malicious attacks, the location error of GPS positioning is rarely discussed. Interested readers may review the method for error rate analysis of GPS receiver systems [10]. There are two major aspects that should be considered in the error rate analysis of GPS receiver systems: (1) finding error sources including deterministic error and non-deterministic error; and (2) establishing a mathematical error model to analyze data, like Gauss-Markov (GM) and auto-regressive (AR) models. We assume that the localization accuracy of the embedded GPS receivers or other positioning chips is acceptable for most of the applications.

The sensor nodes have mobility and move according to the RWP model [11]. In this model, each node randomly chooses a destination point (waypoint) in the sensing field, and moves toward it with aspeed randomly selected from a predefined interval. After reaching the destination, the node stays static for a random time and then moves again according to the same rule. In general, the models used in this paper are the same as the ones in most of the existing works.

### 2.2. Security Model

Identity-based cryptography, which is elaborated in [1,5,12,13], is a rationale in this paper. Each node is pre-allocated an unique identity ${\text{ID}}_{i}$, one public and private key pair, and a hash function H(∙). We assume that an authentication node could be captured and replicated in the network. These replicas of the compromised nodes have all the legitimate key materials of the captured nodes. This means that the replicas of the compromised nodes could access the network by pretending to be legitimate nodes. However, though nodes can be replicated, the new legitimate node IDs could not be created. It is also impossible to decrypt the contents of the network by capturing nodes’ key materials.

## 3. The Security Mechanism

The core work of the mechanism is publishing and verifying the node positions. The public key of the location-based key (LBK) is composed of the identity and the location of a node. The private key is generated with identity-based cryptography by the trusted key generation entity, e.g., the sink node, of the network. The effective range of a location-based key is a circular area centered at the location included in LBK with a radius of ∆. The circular area is called a moving security area (MSA). The location-based key is valid if a node is within its own MSA. In this situation, mutual authentications and key negotiations in nodes can be ensured. Nodes apply for updates to their location-based keys with the sink node when they move out of their own MSAs. The sink node needs to verify the validity of the requests.

The proposed scheme consists of local detection (LD) and overall detection (OD). LD is a detection algorithm which is used within MSA. In contrast, OD is used out of MSA. LD includes level 1 detection (LD-1) and level 2 detection (LD-2). LD-1 is mainly used in nodes’ one-hop neighbors and LD-2 is used in nodes’ two-hop neighbors.

### 3.1. Local Detection

The difference between LD-1 and LD-2 is whether nodes exchange their neighbor lists or not. To put it simply, the replicas of the compromised nodes could be detected directly in the communication range of nodes in LD-1, while in LD-2, replication attacks detection involves nodes’ two-hop neighbors, which could be detected by exchanging their neighbor lists.

#### 3.1.1. LD-1 Mechanism

*Preparation phase:* Node *u* is preallocated $\text{H}(ID_{u})$ and $s_{1}H\left(ID_{u}\right)$ before it is deployed in the network. $\text{H}(ID_{u})$ and $s_{1}H\left(ID_{u}\right)$ denote its identity-based public key and identity-based private key, respectively. $s_{1}$ is the master key materials which are used in identity-based keys. The identity-based public key of sink node $\text{H}(ID_{\text{SN}})$ is stored in node *u*. Node *u* can get the location-based public key $\text{H}(ID_{u}\left|\left|LOC_{u}^{T_{u}}\right|\right|{∆}_{u})$ and the location-based private key $s_{2}\text{H}(ID_{u}\left|\left|LOC_{u}^{T_{u}}\right|\right|{∆}_{u})$ when it is deployed in the network; here ″||″ denotes the concatenation of message. $LOC_{u}^{T_{u}}$ denotes the location of node *u* at time ${\text{T}}_{u}$. $s_{2}$ is the master key materials which is used in location-based keys. ${∆}_{u}$ denotes the radius value of node *u*’s MSA. It will change consistently when node is in different detection levels. Different values of ∆ could have an effect on network security. It will be detailed in Section 4.

*Authentication Phase:* Node u’s MSA is a circular area which is to $LOC_{u}^{T_{u}}$ as the center and a radius of ${∆}_{u}$. All the keys of node *u* are legitimate when *u* is within its own MSA. It means that node *u* could communicate with other nodes in the MSA by using the authenticated keys. When node *u* wants to communicate with its neighbors, the authentication processes will be executed as follows.

u→*: ($SIG_{LK_{u}}(loc_{u}^{t_{u}}∥{\text{n}}_{u}),\left(loc_{u}^{t_{u}}∥{\text{n}}_{u}∥{\text{ID}}_{u}∥{\text{LOC}}_{\text{u}}^{{\text{T}}_{u}}∥{∆}_{u}\right)$)v→u: ($SIG_{LK_{v}}(loc_{v}^{t_{v}}∥{\text{n}}_{v}∥{\text{n}}_{u}∥1),\left(loc_{v}^{t_{v}}∥{\text{n}}_{v}∥{\text{n}}_{u}∥{\text{ID}}_{v}∥{\text{LOC}}_{\text{v}}^{{\text{T}}_{v}}∥{∆}_{u}\right)$)u→v: $SIG_{LK_{u}}\left({\text{n}}_{u}∥{\text{n}}_{v}∥2\right)$

Node *u* signs its current location ${\text{loc}}_{\text{u}}^{{\text{t}}_{u}}$ at time ${\text{t}}_{u}$ and a random number ${\text{n}}_{u}$ by using its location-based key $LK_{u}$, and then broadcasts the signed messages. Node *u*’s ID and ${\text{LOC}}_{\text{u}}^{{\text{T}}_{u}}$ are also included in the messages. ${\text{LOC}}_{\text{u}}^{{\text{T}}_{u}}$ is the center of MSA. It is also the location where it is authenticated when node *u* applies for getting or updating the location-based keys. $SIG_{LK_{u}}$ denotes the messages that are signed with location-based private keys of node *u*. Assume that node *v* is one of the node *u*’s neighbors. After node *v* receives the broadcasting messages from node *u*, the public key $LK_{u}$ of node *u* will be generated by using ${\text{ID}}_{u}$ and ${\text{LOC}}_{\text{u}}^{{\text{T}}_{u}}$ in the messages and the value of ∆. Then node *v* will verify the signed message of node *u* by using $LK_{u}$. It will also check whether node *u* is within its own MSA or not. If node *u* is out of its security area, node *v* would alert node *u* to update its location-based key. In contrast, node *v* would test whether $\frac{\left|{\text{LOC}}_{\text{u}}^{{\text{T}}_{u}}-{\text{loc}}_{\text{u}}^{{\text{t}}_{u}}\right|}{\left|T_{u}-t_{u}\right|}\leq v_{max}$ is established. If the inequation is established, node *v* would test whether $\left|{\text{LOC}}_{\text{u}}^{{\text{T}}_{u}}-{\text{LOC}}_{\text{v}}^{{\text{T}}_{v}}\right|\leq \left|{∆}_{u}+{∆}_{v}+R\right|$ is established. If the inequation is not established, it means that node *u* is not a legitimate node. In this situation, node *v* discards the request from node *u*, signs the message and broadcasts the alert message that node *u* is a malicious node. If all of the inequations are established, node *v* would sign the message including its current location $loc_{v}^{t_{v}}$, a random number ${\text{n}}_{v}$ that is generated by node *v*, a random number ${\text{n}}_{u}$ and a number 1. Next node *v* sends its own ID and the authenticated location ${\text{LOC}}_{\text{v}}^{{\text{T}}_{v}}$ to node *u*. Similarly, node *u* verifies the validity of node *v* using the same processes. Node *v* will receive a signed message $SIG_{LK_{u}}\left({\text{n}}_{u}∥{\text{n}}_{v}∥2\right)$ from node *u* after passing authentication. Then it will regenerate a signature to compare with the signed message that was just received. If two signatures are same, node *v* would admit node *u* is a legitimate node and an authentic neighbor.

#### 3.1.2. LD-2 Mechanism

In LD-2, nodes exchange neighbor lists which contain information of neighbor nodes when they encounter them in the network. For ease of description, we assume there are two nodes *u* and *v* in the network. When node *u* find node *v* in its communication range, it stores node *v*’s location information, ID and the current time denoted by ($LOC_{v}^{T_{v}}∥loc_{v}^{t_{v}}∥ID_{v}$). At the same time, node *v* does the same thing like node *u* does. Thus nodes can collect enough information about their two-hop neighbors. If there exit contradictions, nodes would sign alert messages and broadcast them to the network. Other details such as authentication processes are similar to LD-1.

### 3.2. Overall Detection

Overall detection (OD) is a centralized algorithm. In this mechanism, the key materials of node *u* are legitimate as long as node *u* is within its own MSA which has ${\text{LOC}}_{\text{u}}^{{\text{T}}_{u}}$ as the center and a radius of ${∆}_{u}$. The location-based key of node *u* is invalid when node *u* moves out of its own MSA. In this case, node *u* receives some requirements about updating keys from its neighbors and then applies for a new location-based key with the sink node.

Specific updating-related processes are as follows. Firstly, node *u* broadcasts the request about updating its location-based key. The validity of node *u* is authenticated by at least *t* neighbors. These neighbors are called witness nodes. After that, *t* witness nodes will test whether $\left|LOC_{u}-loc_{u}\right|\geq {∆}_{u}$ and $\frac{\left|LOC_{u}-loc_{u}\right|}{\left|T_{u}-t_{u}\right|}\leq {\text{v}}_{max}$ are established or not. If the inequations are established, these witness nodes will sign the request messages by using their own location-based keys: $({\text{SIG}}_{LK_{i}}\left(loc_{u}^{t_{u}}∥ID_{u}\right),\;\;\;$$\left(loc_{u}^{t_{u}}∥ID_{u}∥ID_{i}∥LOC_{i}^{T_{i}}∥{∆}_{i}\right))$, and then send the messages to the sink node. In contrast, the alert messages will be sent to the network to tell other nodes that node *u* is a malicious node.

Malicious nodes could not collude with each other to defraud a new legitimate location-based key unless these *t* witness nodes are all captured in the communication range of node *u* at the same time. The value of parameter *t* depends on the average number of node *u*’s neighbors. Here we set *t* = 3 to ensure higher probability of successfully updating keys. When the sink node receives t request messages marked with the same ID, it confirms the validity of *t* witness nodes by verifying whether the signed messages are legitimate. Next, the sink node further confirms the validity of node *u* by checking the continuity of node *u*’s movement path. The messages, which are composed of node ID, location information and current time of each node in network, are recorded in the sink node. Next, the sink node tests whether the inequations $\left|LOC_{u}-loc_{u}\right|\geq {∆}_{u}$ and $\frac{\left|LOC_{u}-loc_{u}\right|}{\left|T_{u}-t_{u}\right|}\leq {\text{v}}_{max}$ are established; here the value of parameter ${∆}_{u}$ depends on the area security needs. If either of two inequations is not established, there are malicious witness nodes colluding to defraud the location-based keys. That is because the legitimate witness nodes should have tested these inequations and ensured that node *u* is a legitimate node. However, this is just an extreme case, and only happens when there are too many nodes captured by adversaries so that there is no legitimate node in some local areas. If both of the two inequations are established, the sink node will set $LOC_{u}^{T_{u}}\leftarrow loc_{u}^{t_{u}}$ and ${∆}_{u}\leftarrow \;∆$. The updating key of node *u* will be encrypted with node *u*’s identity-based public key and signed by the sink node’s identity-based private key $\left({\text{E}}_{IK_{u}}\left({\text{SIG}}_{IK_{SN}}\left({\text{LOC}}_{\text{u}}^{{\text{T}}_{u}}∥{∆}_{u}\right),\left({\text{LOC}}_{\text{u}}^{{\text{T}}_{u}}∥{∆}_{u}∥{\text{ID}}_{SN}\right)\right)\right)$. Here ${\text{E}}_{IK_{u}}\left(\cdot \right)$ denotes that the message is encrypted with node *u*’s identity-based key, so a new location-based only could be decrypted by node *u* because only node *u* has the corresponding identity-based private key.

There is a situation in which node *u* has moved out of its own MSA but could not be authenticated because the number of witness nodes is less than *t*. The random deployment of network nodes can be modeled as a spatial homogeneous Poisson point process [14]. Hence, the probability that the witness nodes number less then *t* is:

(1) $$\text{P}\left(\left|n\right|<t\right)=\overset{t-1}{\underset{i=0}{\sum }}\frac{{\left(\rho \pi R^{2}\right)}^{i}}{i!}e^{-\rho \pi R^{2}}$$

and the probability that the number of witness nodes is greater than *t* is:

(2) P(|n|≥t)=1−P(|N|<t)

The factor ρ denotes node density; here $\text{ρ}=\frac{\left|{\text{N}}_{\text{all}}\right|}{\text{A}}$ ($\left|{\text{N}}_{\text{all}}\right|$ denotes the total number of nodes and A denotes the area of the deployment region). We assume that the maximum distance is ξ when node *u* has moved out of MSA but still does not update its location-based private key. The time interval that node *u* applies for updating key once more is ∆t. Assume that node *u* has sent n requests about updating the key when it receives a new key. This means the probability that u still does not update its key when it is in a position where is ${∆}_{\text{u}}+\text{ξ}$ away from the MSA is:

(3) $${\text{ P}}_{u}={\left(\text{P}\left(\left|n\right|<t\right)\right)}^{n-1}(\text{P}\left(\left|n\right|\geq \text{t}\right)={\left(\overset{t-1}{\underset{i=0}{\sum }}\frac{{\left(\rho \pi R^{2}\right)}^{i}}{i!}e^{-\rho \pi R^{2}}\right)}^{\text{n}-1}(1-\overset{t-1}{\underset{i=0}{\sum }}\frac{{\left(\rho \pi R^{2}\right)}^{i}}{i!}e^{-\rho \pi R^{2}})$$

The value of the probability P(|n|<t) always approaches zero unless the nodes in networks are very sparse. That is to say, nodes would update their location-based private keys immediately when they move out of MSA, because the probability that there are enough witness nodes in nodes’ communication range is actually substantial. In other words, ξ→0.

## 4. Analysis

In this section, we evaluate the performance of our mechanism. We build models and analyze the performance of the proposed scheme based on a two-dimensional mobile sensor network where sensor nodes freely move throughout the deployed area. The unit disk graph (UDG) communication model is adopted as the analysis component of most of the literature about sensor networks [6,15,16]. All direct communication links between sensor nodes are assumed to be bidirectional. This communication model is common in the current generation of sensor networks [17]. We mainly focus on how to mitigate malicious attacks, especially how to improve detection performance for replication attacks in the commonly used communication models.

The communication range of each node is denoted by *R*. Since a perfect circular communication cutoff cannot be expected in a real environment, the value *R* actually defines the circular area where all the nodes can be reached properly. Although two nodes may still communicate over a distance greater than *R*, we ignore these cases to make the value of *R* strict enough to ensure the reliability of neighborhood communication.

We assume that the sink node transmits with higher power than sensor nodes and hence has a sink-to-sensor communication range of L > R. Note that to achieve a communication range ratio L/R, the sink needs to transmit with power $P_{S}={\left(L/R\right)}^{\gamma }(P_{n}/G)$, where $P_{n}$ is the sensor transmission power, γ is the signal attenuation factor and G is the directivity gain. Given that sensors are very low power devices, the higher transmission power assumption on the sink side is reasonable. A typical sensor has a communication range from 3 m to 30 m with a transmission power of $P_{n}$ = 0.75 mW. Hence, the sink needs to transmit with a power $P_{S}$ = 75 mW to achieve a communication range ratio L/R = 10 when γ = 2.

We assume that GPS systems or localization schemes have been applied to the network such that all nodes know their own positions. The core idea of the proposed scheme is to identify the authenticity of the location claimed by a node with the aid of the benign witness nodes. Especially when a replication attack is locally detected, it is a crucial prerequisite that there are witness nodes in a specific region suitable for observation.

Recall the statistics theory for spatial node distribution. The random deployment of the network nodes can be modeled as a spatially homogeneous Poisson point process [14]. The random placement of the nodes with a density $\rho_{N}=|N|/A$ is equivalent to a sequence of events following a homogeneous Poisson point process of rate $\rho_{N}=|N|/A$. Given that N events occur in area $\rho_{N}=|N|/A$, these events are uniformly distributed within $\rho_{N}=|N|/A$. Let S denote the set of nodes located in area α, giving us:

(4) $$P(|S|=k)=\frac{{\left(\rho_{N}\alpha \right)}^{k}}{k!}e^{-\rho_{N}\alpha }$$

Using Equation (2), we can deduce the probability that there are at least k nodes within area α:

(5) $$P(|S|\geq k)=1-\sum_{i=0}^{k-1}\frac{{\left(\rho_{N}\alpha \right)}^{i}}{i!}e^{-\rho_{N}\alpha }$$

### 4.1. Security Analysis

#### 4.1.1. Replication Attack

Since nodes need authentication keys to communicate with other nodes in our mechanism, a variety of external attacks could be resisted effectively because those nodes cannot provide a legitimate key to access the network. We detail how the mechanism MSA resists replication attack. The influences will be shown by adjusting the value of ∆.

$\left|{\text{uu}}^{\prime }\right|<\text{R}$: If the compromised node *u* and the replica of the compromised node *u*’ collude in claiming the same position and carefully choosing one of them to transfer messages, the other meanwhile keeps silent to escape the detection. In this case, the replica of the compromised node is hard to detect because of the accuracy limit of the location verification. On the contrary, malicious nodes could be detected easily when the compromised node and the replica of the compromised node claim different positions. In a real situation, the replicas of the compromised nodes would be deployed in a large region to get more secret information. Hence we will move on to the next situation.

**Figure 1:**
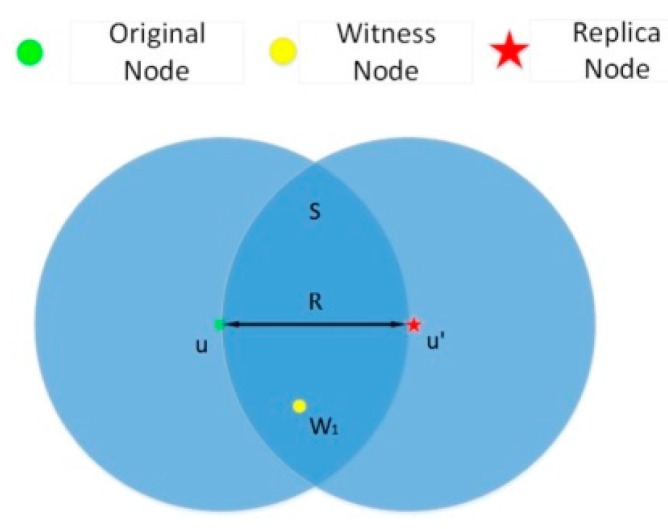
An example of detecting a replication attack by using LD-1.

$\text{R}\leq \left|{\text{uu}}^{\prime }\right|<2\text{R}$: Figure 1 depicts an overlapped area S between ${\text{MSA}}_{\text{u}}$ and ${\text{MSA}}_{{\text{u}}^{\prime }}$. Replication attacks will be detected if there is at least one witness node in area S. MSA_LD-1 works in this situation and we set the value of ∆ to R. The witness nodes could detect contradiction that two nodes marked with the same ID are in different positions at the same time by communicating with both of them.

According to Equations (1) and (2), the following equation can be derived:

(6) $$\text{P}\left(\left|n\right|\geq 1\right)=1-\text{P}\left(\left|n\right|<1\right)=1-e^{-\rho S}$$

**Figure 2:**
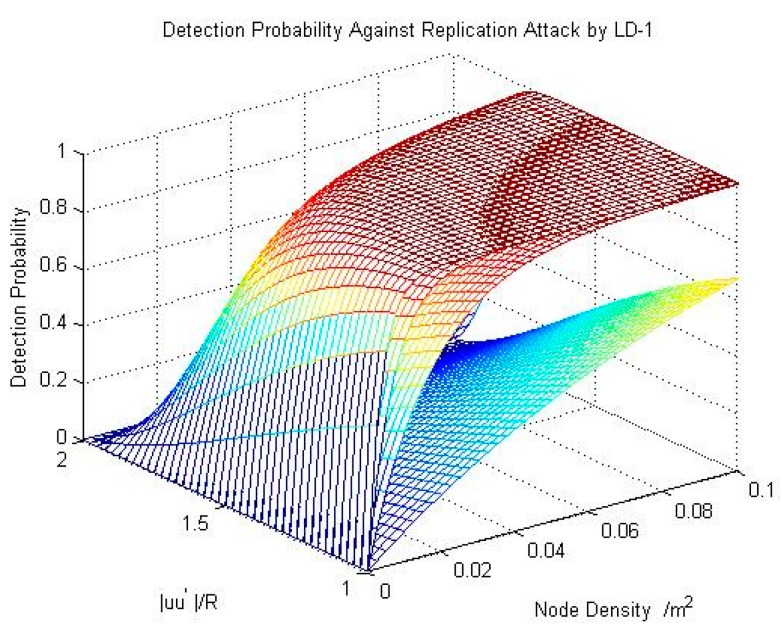
The result of detection probability for resisting replication attacks by using LD-1.

Equation (6) reflects the probability that there is at least one node in area S. Figure 2 shows the detection probability.

The probability is related to the probability that there exist nodes in the overlapped area. That is to say, it is related to nodes’ communication range R and nodes’ density ρ. As shown in Figure 2, the value range of ρ is from 0 to 0.1. ρ denotes the number of nodes per unit area. We set the values of R as 15 m, 11 m, 7 m and 3 m, respectively. With a smaller communication range, a lower detection probability follows. The probability is higher when the density of nodes is increased.

**Figure 3:**
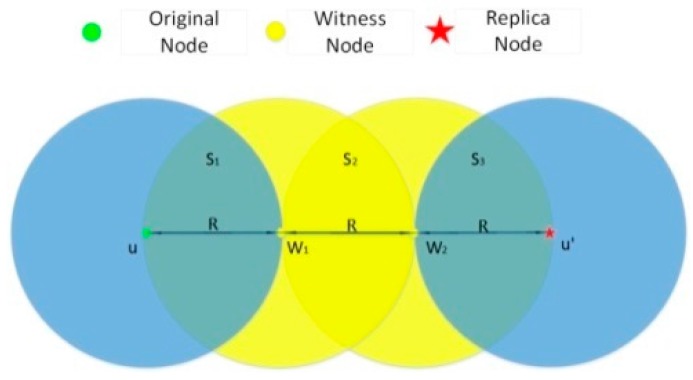
The model of detecting replication attack by using LD-2.

$2\text{R}\leq \left|{\text{uu}}^{\prime }\right|<3\text{R}$: In this case, we could use LD-2 to detect the replicas of the compromised nodes, as shown in Figure 3. Here, we set ∆ =1.5R.

Figure 3 depicts an extreme situation in which $\left|{\text{uu}}^{\prime }\right|=3\text{R}$. In this case, witness node ${\text{w}}_{1}$ has the information of u and meanwhile ${\text{w}}_{2}$ has the information of ${\text{u}}^{\prime }$. When these two witness nodes encounter each other, they exchange their neighbor lists and find the contradiction that two nodes marked with the same ID are in different positions at the same time. Thus the attack is detected. Next, the probability is calculated.

**Figure 4:**
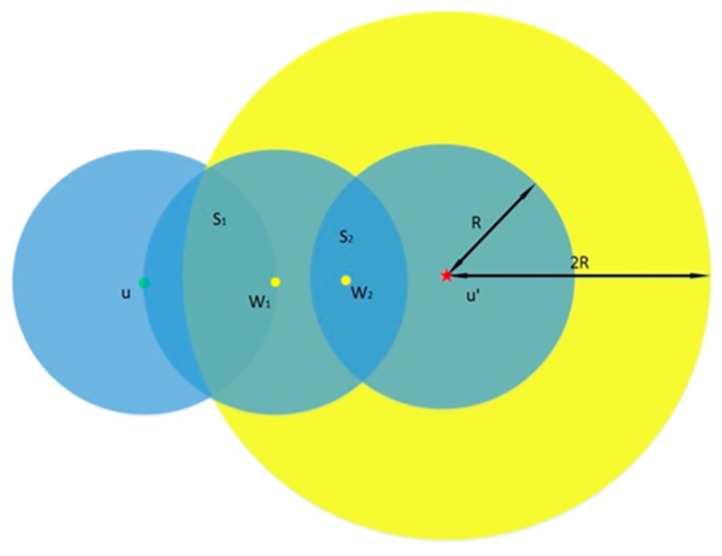
An example to illustrate that using the LD-2 detection model fights against a replication attack.

As shown in Figure 4, the critical distance between ${\text{w}}_{1}$ and ${\text{u}}^{\prime }$ is 2R. ${\text{S}}_{1}$ is the overlapped area between the first small circular area and the biggest circular area. In this overlapped area, ${\text{w}}_{1}$ can communicate with u and ${\text{w}}_{2}$ at the same time. ${\text{S}}_{2}$ is the overlapped area between ${{\text{w}}_{1}}^{\prime }\text{s}$ communication range and the circular area with center point ${\text{u}}^{\prime }$. In the area ${\text{S}}_{2}$, ${\text{w}}_{2}$ can communicate with ${\text{w}}_{1}$ and ${\text{u}}^{\prime }$ at the same time.

In this model, the detection probability is:

(7) $$\text{P}=\left(1-e^{-\rho S_{1}}\right)*\left(1-e^{-\rho S_{2}}\right)$$

${\text{P}}_{1}$ represents the probability there is at least one node in ${\text{S}}_{1}$. ${\text{P}}_{2}$ denotes the probability that there is at least one node in ${\text{S}}_{2}$. When ${\text{w}}_{1}$ in ${\text{S}}_{1}$, meanwhile ${\text{w}}_{2}$ in ${\text{S}}_{2}$, the witness node ${\text{w}}_{1}$ encounters ${\text{w}}_{2}$ and they exchange their neighbor lists so that they can detect replication attacks and broadcast the warning messages.

**Figure 5:**
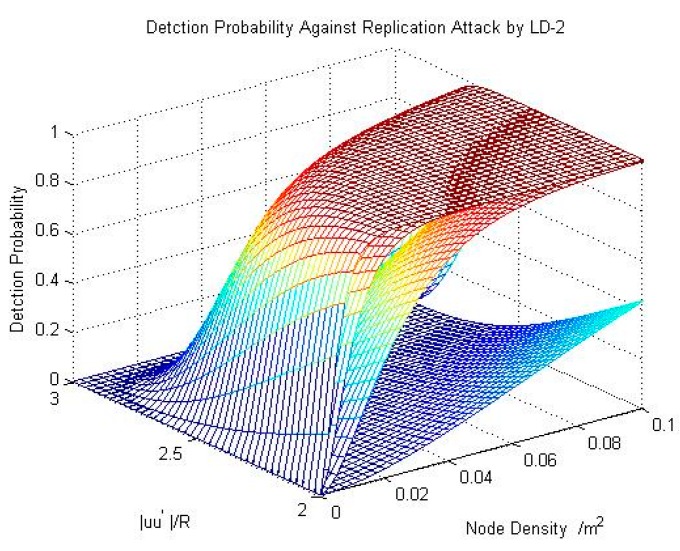
The result of detection probability for resisting a replication attack by using LD-2.

We set $\left|{\text{uw}}_{1}\right|=\text{R}$ to make the value of ${\text{S}}_{1}$ the largest, and then we can obtain the upper limit of the detection probability as shown in Figure 5.

$\left|{\text{uu}}^{\prime }\right|\geq 3\text{R}$: Since the value of ∆ is not high enough in the above cases, nodes will update their location-based private keys too frequently, especially when nodes move quickly. On the other hand, more frequently updating ensures higher security. Hence the smaller value of ∆ should be set in some areas required higher security or some lower speed mobile models. In most common cases, we would like to set $∆\;={\text{ V}}_{\text{max}}\;\times \text{T}$. T represents a time period and ${\text{V}}_{\text{max}}$ denotes the maximum speed of nodes.

We could get the upper limit of detection probability by employing the analysis method in [6]. The external square of MSA is partitioned into a number of regular grids. The side length of each grid is $\frac{\sqrt{2}}{2}\text{R}$. Thus any two nodes could communicate with each other within one grid. We assume that there are q grids in the largest MSA and each node has d neighbors. Let W and ${\text{W}}^{\prime }$ denote the set of witness nodes of compromised node u and the set of witness node of replica ${\text{u}}^{\prime }$, respectively. Event ${\text{W}}_{\text{i}}$ denotes assigning d elements in set W into i different grids. We have that:

(8) $$\text{P}\left[{\text{W}}_{\text{i}}\right]=\;\frac{\left(\begin{array}{c} \text{q}\\ \text{i} \end{array}\right)\text{i}!{\text{i}}^{\text{d}-\text{i}}}{{\text{q}}^{\text{d}}}=\;\frac{\text{q}!{\text{i}}^{\text{d}-\text{i}}}{\left(\text{q}-\text{i}\right)!{\text{q}}^{\text{d}}}$$

Event з denotes that there are elements in set W but there are not elements in set W’ in i grids. The probability of з is derived:

(9) $$\text{P}\left[{\text{з|W}}_{\text{i}}\right]={\left(\frac{\text{q}-\text{i}}{\text{q}}\right)}^{\text{d}}\cdot \text{P}\left[{\text{W}}_{\text{i}}\right]$$

$\text{P}\left[{\text{D}}_{\text{j}}\right]$, which denotes the probability that attacks are detected in the first j rounds, is derived:

(10) $$\text{P}\left[{\text{D}}_{\text{j}}\right]=1-(\overset{\text{d}}{\underset{\text{i}=1}{\sum }}{\left(\frac{\text{q}-\text{i}}{\text{q}}\right)}^{\text{d}}\cdot \text{P}\left[{\text{W}}_{\text{i}}\right]{)}^{\text{j}}$$

$\text{j}=\;\frac{\text{T}}{∆\text{t}}$, and ∆t denotes a short time gap.

The processes for deriving these equations are simplified. If readers are interested in the deduction process, please see the references [6].

It is a corollary that contradictions are detected by exchanging neighbor lists within ordinary nodes [3,6,15]. If replicas of the compromised nodes are deployed out of MSAs, their location-based private keys are invalid. When the range is limited to a smaller area, the MSA, there is a higher probability that witness nodes encounter replicas of the compromised nodes. What is more, the farther away the replicas of the compromised nodes, the easier replication attacks are detected in OD. The legality of nodes is verified by the sink node when these nodes ask to update their location private keys.

#### 4.1.2. Sybil Attack

In a Sybil attack, a single node presents multiple identities to other nodes in the network. It is only reasonable to expect a node to accept but a single set of coordinates from each of its neighbors, but by using the Sybil attack an adversary can be in more than one place at once.

The prerequisite for a node to pass authentication is to own a legitimate location-based private key. Location-based key management for mobile networks is implemented to mitigate a Sybil attack. With our scheme in place, when a malicious node intends to impersonate a legitimate node, it does not have the authentic LBK and, thus, cannot successfully finish mutual authentication with other legitimate nodes. For the same reason, a malicious node cannot claim forged IDs and/or locations without being detected. Therefore, the Sybil attack is effectively defeated.

#### 4.1.3. Wormhole Attack

A wormhole attack [18] is a notorious attack against WSN routing protocols that is difficult to withstand. In a wormhole attack, instead of compromising any node, collaborative adversaries first create a wormhole link, essentially an out-of-band and low-latency channel, between two distant network locations. They then tunnel routing messages recorded at one location via the wormhole link to the other, leading to chaos in the routing operations. A variety of attacks could be launched by attracting a large number of network flows via the channel.

To identify a wormhole attack, a node must verify if the message originated from its direct neighbor. Since location-based authentication is built in our scheme, the message authentication code (MAC) is attached to the data from a legitimate node. The message includes the data from the node concatenated with its location-based public key. The MAC is generated by signature of the message digest with the location-based private key. When a node receives a message, it first examines whether the public location-based key is from an authenticated direct neighbor node and will discard those tunneled from distant locations. Next it verifies the MAC with the location-based public key included in the message and will discard those cannot pass the verification. Thus the message and its generated location are simultaneously verified.

In our mechanism, because only messages from legitimate neighbors which locations are verified will be accepted by nodes, the wormhole attack is effectively resisted.

### 4.2. Overhead Analysis

In our mechanism, each node should update location-based private key when it moves out of its own MSA. The expected numbers of nodes, which update their location-based keys in interval T, are evaluated. Let ${\text{E}}_{\text{update}}$ denote the expected numbers. First of all, the upper limit of the expected numbers will be derived. We assume that all nodes move at the maximum speed ${\text{V}}_{\text{max}}$. In an interval ${\text{T}}_{\text{i}}$, nodes are deployed at different positions within their own MSA at a certain time. Every node’s moving distance is no more than ∆ in next interval T. Since the moving directions of nodes are chosen randomly, nodes update their location-based keys when they move out of their own MSAs. The probability for updating location-based keys is derived as follows:

(11) $$P_{update}\;=\;1-\frac{S}{\pi {∆}^{2}}$$

**Figure 6:**
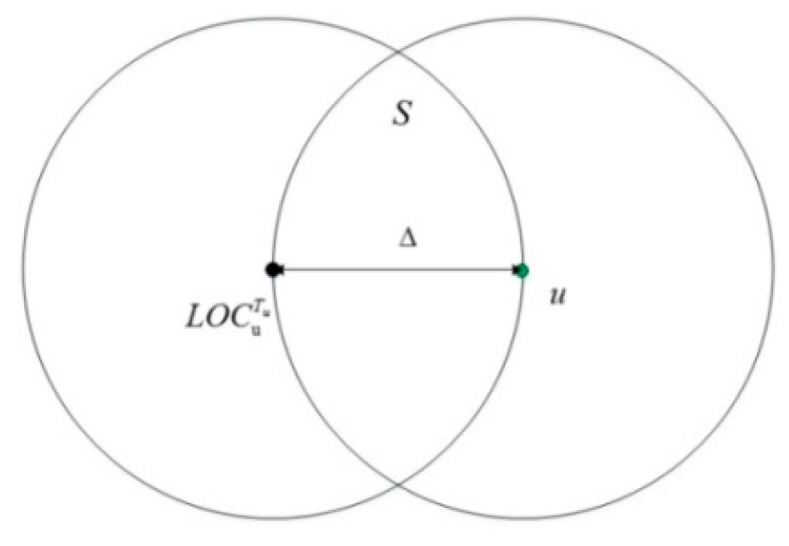
An example of updating the location-based keys in an extreme case.

If node u is within its own MSA, the location-based key is always valid. Let $\text{π}{∆}^{2}$ denote the set of regions that node u can get to in next interval T. As shown in Figure 6, S denotes the overlapped region. The probability for updating the location-based key is the probability that node u moves out of the MSA.

We use binomial distribution to compute expected numbers. Then ${\text{E}}_{\text{update}}$ can be calculated by equation ${\text{E}}_{\text{update}}=\text{N }\times {\text{ P}}_{\text{update}}$. Let N denote the total number of nodes in the network; the value of ${\text{E}}_{\text{update}}$ is 0.609N by calculation. This means that 60.9% of nodes will update their location-based private keys in interval T when all nodes are deployed at the boundary of their own MSAs and move at the maximum speed ${\text{V}}_{\text{max}}$. It is also the largest value of ${\text{E}}_{\text{update}}$.

We assume that the density of nodes is a constant ρ. In general, node u is randomly deployed in its own MSA. The radius of each MSA is divided into k equal parts, that is to say, each MSA is partitioned into a circular area and k-1 concentric ring areas. We assume that each node is deployed at the boundary of one of the k areas and moves at the speed ${\text{V}}_{\text{max}}$. Let ${\text{N}}_{\text{i}}$ denote the number of nodes in the i-th area and ${\text{A}}_{\text{i}}$ denote the area of the i-th region. Equation (12) calculates the number of nodes in each area:

(12) $$N_{i}=\;\rho \times A_{i}$$

N denotes the total number of nodes in MSA. Then, we can derive:

(13) $$\text{N}=\;\overset{k}{\underset{i=1}{\sum }}N_{i}=\overset{k}{\underset{i=1}{\sum }}\left(\rho \times A_{i}\right)$$

**Figure 7:**
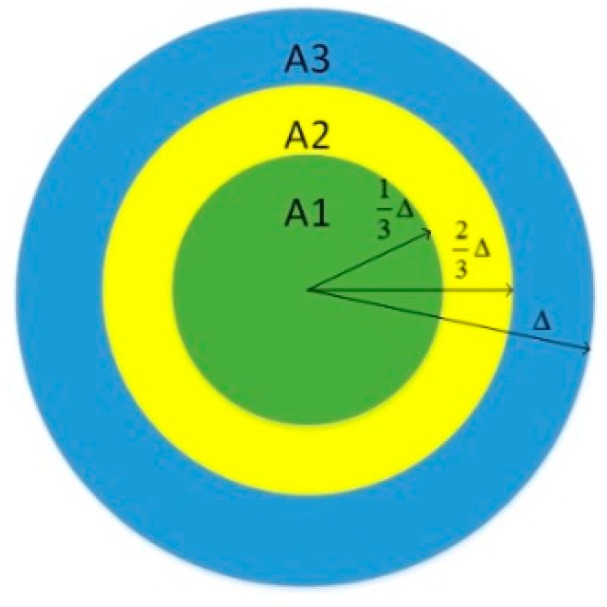
An example of how to divide MSA when k = 3.

**Figure 8:**
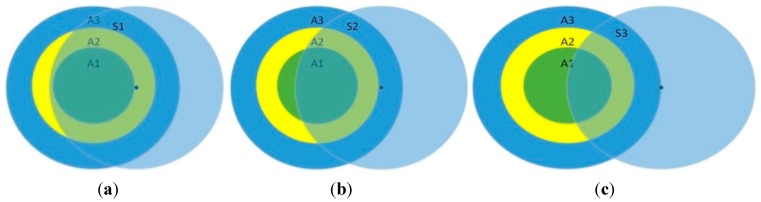
An example of how nodes are deployed at the boundary of area (**a**) A1; (**b**) A2 and (**C**) A3.

Figure 7 is an example illustrating how to divide MSA when k = 3. There is no doubt that nodes must be deployed in one of these 3 divided areas. We assume that all nodes are deployed at the boundary of their areas, as shown in Figure 8a–c. ${\text{S}}_{1}$, ${\text{S}}_{2}$ and ${\text{S}}_{3}$ all denote the overlapped area between the region that nodes can get into in the next time interval and the current MSA.

Equation (14) calculates the expected number of key-updating nodes:

(14) $$\text{E}=\;\overset{k}{\underset{i=1}{\sum }}P_{i}\times N_{i}$$

**Figure 9:**
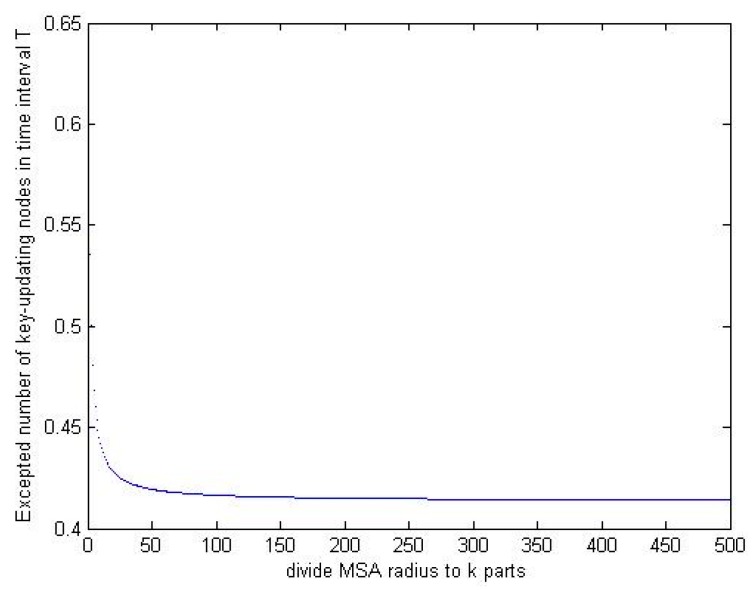
The upper limit of the expected numbers of key-updating nodes.

In the equation, ${\text{N}}_{\text{i}}$ denotes the number of nodes in the i-th area, and ${\text{P}}_{\text{i}}$ denotes the corresponding key-updating probability. With the value of k increasing, MSA will be finely partitioned, hence the expected number E will decrease gradually and eventually keep stable at 0.41 N. Figure 9 shows the upper limit of the expected numbers of key-updating nodes.

It seems that there are many nodes need update their location-based private keys in one period T. However the expected number is computed in the extreme case that all nodes are updated at the boundary of their own MSAs and move at the maximum speed, the number of the key-updating nodes will be smaller in actuality.

Next we evaluate the overhead of our mechanism in terms of communication, computation and storage compared with some existing schemes. The comparison is showed in Table 1. As LD-1 is local detection, nodes only need communication with their neighbors and update their private keys when needed. Hence the communication and computation overhead are O(1). Nodes do not need to store other information except for their related key materials, thus the storage cost of LD-1 is O(1).

LD-2 is also a local detection, so the communication and computation overhead are O(1). Neighbor lists need to be exchanged between every node and their neighbors, so the storage overhead is O(N), that is because in the extreme case, each node need save the information about other N-1 nodes.

The overall detection OD is executed by the sink node. Since the sink node usually has higher computation capability and larger storage space [1,2,3,19], some detecting tasks could be transferred to the sink node to ensure better energy balance. When node u applies for updating its private key at a certain position in the monitory region, sink node will receive the authentication messages from t witness nodes. The sink node will also send the new key to node u after ensuring the messages are legitimate by checking the authentication messages. In the extreme case, there are 0.41 nodes need update their private keys. There are t + 1 messages which will be transmitted when private keys are updated, including receiving t authentication messages and sending one updated key. Hence the communication overhead is O (0.41(t + 1) N). The computation cost is O(1) because the sink node needs to store information from all nodes. It is important to note that the communication overhead in our mechanism OD is global, but in other papers it usually means the cost of one single node. For example, the overall communication overhead of TDD is $\text{O}\left(\text{N}\sqrt{\text{N}}\right)$. By contrast, our mechanism has less overhead.

**Table 1:** Overhead of different schemes, CommO: Communication overhead; CompO: Computation overhead; SO: Storage overhead; AA: Against attacks: SA: Sybil attack; RA: Replication attack; WA: Wormhole attack; SNT: Supported network type.

Scheme	CommO	CompO	SO	AA	SNT
LBK [1]	—	—	—	SA/RA/WA *etc.*	Static Networks
TDD [3]	$\text{O}\left(\sqrt{N}\right)$	O(1)	O(N)	RA	Mobile Networks
SDD [3]	O(1)	O(1)	O(N)	RA	Mobile Networks
HIP [5]	O(d^2^h)	O(d^3^h)	O(d^2^h + dh)	RA	Mobile Networks
HOP [5]	O(d^2^h)	O(d^3^h)	O(d^2^h + dh)	RA	Mobile Networks
XED [15]	O(1)	O(1)	O(N)	RA	Mobile Networks
EDD [15]	O(1)	O(1)	O(1)	RA	Mobile Networks
Patrol Detection [20]	O(n) & $\text{O}\left(\text{n*}\sqrt{k}\right)$	O(1)	—	RA	Static & Mobile Networks
MSA_LD-1	O(1)	O(1)	O(1)	SA/RA/WA *etc.*	Mobile Networks
MSA_LD-2	O(1)	O(1)	O(N)	SA/RA/WA *etc.*	Mobile Networks
MSA_OD	O(0.41(t+1)N)	O(1)	O(N)	SA/RA/WA *etc.*	Mobile Networks

## 5. Conclusions

A new security mechanism, MSA, is proposed in this paper. Compared with some related research, location-based keys are bound with the IDs of the mobile nodes to mitigate malicious attacks, such as replication attacks, Sybil attacks and wormhole attacks. The parameter ∆, which is the radius of the MSA, is a user-defined value that can be set to satisfy different security levels. Local detection is distributed and performed by the ordinary nodes inside the MSA without the participation of the sink node. However, in overall detection, detection tasks are transferred to the sink node and performed along with the key updating operations. Simulation results reveal that proposed mechanism performs well when it resists malicious attacks and displays better results in terms of detection accuracy and energy balance.
